# ASSOCIATIONS BETWEEN SELF-RATED HEALTH, COGNITIVE FUNCTION, RISK OF COGNITIVE IMPAIRMENT, AND DEMENTIA

**DOI:** 10.1093/geroni/igae098.3037

**Published:** 2024-12-31

**Authors:** Kayla Garner, Eileen Graham, Kathryn Jackson, William Chopik, Emorie Beck

**Affiliations:** Northwestern University, Evanston, Illinois, United States; Northwestern University, Evanston, Illinois, United States; Northwestern University, Evanston, Illinois, United States; Michigan State University East Lansing, East Lansing, Michigan, United States; University of California Davis, Davis, California, United States

## Abstract

Psychosocial researchers have long sought to identify individual differences in early and middle adulthood that robustly predict health in later life. Our current pre-registered study builds on these efforts by assessing whether an individual’s subjective perception of their health is related to their cognitive health, both concurrently and later in life. Using the coordinated data analysis (CDA) methodological framework, we have identified 17 longitudinal studies (including 7 from the Gateway to Global Aging network) that had the requisite data to test our models. We conceptually harmonized all study variables and tested identical linear and logistic regression models across all study datasets. Our preliminary results suggest that individuals who have better subjective beliefs about their health have better cognitive function both concurrently and at longitudinal follow-up (ranging from 4 to 27 years after baseline). These associations were moderated by participant gender and age at baseline, and the results were somewhat robust to the inclusion of objective health indicators. We will discuss our results within the context of Lifespan Developmental Theory and the Health Behavior Model. Overall, our study serves to elucidate how subjective perceptions of health may manifest into later cognitive health outcomes, and if subjective perceptions of health could be used as an early risk factor for identifying individuals who may be vulnerable to cognitive senescence in later life.

